# Use of Methylene Blue for Treatment of Severe Sepsis in an Immunosuppressed Patient after Liver Transplantation

**DOI:** 10.1155/2013/203791

**Published:** 2013-05-13

**Authors:** Saravanan Ramamoorthy, Shachi Patel, Eric Bradburn, Zakiyah Kadry, Tadahiro Uemura, Piotr K. Janicki, Riaz Ali Shah, Dmitri Bezinover

**Affiliations:** ^1^Department of Anesthesiology, Penn State Hershey Medical Center, 500 University Drive, H187 Hershey, PA 17033-0850, USA; ^2^Department of Anesthesiology, Penn State Hershey Medical Center, Hershey, PA-17033, USA; ^3^Department of Surgery, Penn State Hershey Medical Center, Hershey, PA-17033, USA; ^4^Department of Transplant Surgery, Penn State Hershey Medical Center, Hershey, PA-17033, USA

## Abstract

Sepsis in the immunosuppressed patient is associated with very high mortality and morbidity. Treatment of sepsis in immunocompromised patients is especially challenging due to an unbalanced systemic inflammatory reaction with subsequent development of profound vasoplegia. Methylene blue (MB) has been successfully used for the treatment of refractory hypotension, but its use has not previously been reported for treatment of sepsis in immunosuppressed patients. The mechanism of MB's action is thought to be due to its inhibitory effect on cGMP-mediated vasodilatation. This case report describes the successful use of MB for treatment of severe septic shock in an immunosuppressed patient after liver transplantation. Hypotension in this patient was refractory to volume repletion and a combination of vasopressors. After MB administration, hemodynamic stability was rapidly reestablished. In the setting of severe sepsis in an immunosuppressed patient, MB should be considered early as a therapeutic option for treatment of refractory vasoplegia.

## 1. Introduction

Sepsis in the immunosuppressed patient is often resistant or unresponsive to conventional pharmacologic therapy and is associated with mortality significantly higher than in the immunocompetent patient. The fulminant course of sepsis in immunosuppressed patients is related to an unbalanced systemic inflammatory response with subsequent development of pharmacologically resistant hemodynamic depression. Severe septic shock with vasoplegia is a devastating complication of sepsis in the immunocompromised patient. Therapy resistant hypotension is thought to be the result of dysregulation of nitric oxide (NO) synthesis. A number of investigations performed in critically ill, although not pharmacologically immunosuppressed patients, have demonstrated the efficacy of methylene blue (MB) administration in reducing plasma NO and restoring hemodynamic stability [1–4]. 

## 2. Case Description

Before the preparation of this paper, written informed consent was obtained from the patient. 

A 64-year-old male patient presented for a routine liver biopsy after orthotopic deceased donor liver transplantation (OLT). The original cause of the patient's liver failure was nonalcoholic steatohepatitis complicated by hepatocellular carcinoma. The patient's past medical history was otherwise not significant. Postoperative immunosuppressive medications included both mycophenolate and tacrolimus. 

Two years after transplantation, the patient underwent a scheduled liver biopsy. A few hours after the procedure, he developed severe right upper quadrant pain which became diffuse and significantly worsened despite repeated administration of morphine sulfate boluses. A CT scan of the abdomen with contrast demonstrated the presence of fluid surrounding the liver as well as fluid accumulation in the lower abdomen and pelvis. There were no other pathological findings. 

The patient's condition continued to rapidly deteriorate. He became febrile to 38.7°C and developed signs of respiratory distress with oxygen saturations below 90% despite 6 L/min oxygen via nasal cannula. On postprocedure day (PPD) 1, the patient became tachycardic to 129 bpm and hypotensive with a blood pressure of 96/68 mmHg. After further deterioration of the patient's cardiopulmonary status, he was intubated and transferred to the Medical Intensive Care Unit (MICU). Due to profound hypotension, intravenous administration of phenylephrine was initiated, first as boluses (100–200 mcg) and later as an infusion of 30 mcg/min. The patient also received an infusion of 0.9% normal saline solution at 200 mL/h. While in the MICU, the patient developed symptoms of peritonitis. CT-guided drainage of the peri-hepatic accumulation demonstrated fluid compatible with an infected biliary drainage. Blood cultures and peri-hepatic fluid cultures were positive for gram-negative *Escherichia coli*. Broad-spectrum antibiotic therapy was initiated with meropenem, ciprofloxacin, vancomycin, tobramycin, as well as caspofungin. Despite antibiotic administration and vasoactive support, arterial hypotension and tachycardia persisted (a low of 88/59 mmHg and heart rate of 135 bpm). Norepinephrine (NE) and vasopressin (VP) infusions were initiated. Infusion rates were titrated up to a maximum of 3 mcg/kg/min of NE and 0.08 units/min of VP without significant improvement in the patient's hemodynamic status. An exploratory laparotomy was performed which demonstrated a bile containing fluid accumulation and a laceration of the liver at the biopsy site. Repair of the laceration and an abdominal lavage were performed.

After surgery, the patient was admitted to the Surgical Intensive Care Unit (SICU) in critical condition. In the SICU, a pulmonary artery catheter was placed for hemodynamic monitoring. The patient displayed a pattern of circulation consistent with sepsis, with a cardiac output of 9.4 L/min, SVR of 298 dyne·s^−1^·cm^−5^, and mixed venous saturation of 78%. In addition to continued vasoactive agent administration, volume therapy was initiated. Overall, 8 liters of crystalloid was administered over the next 10 hours. The patient continued to be hemodynamically unstable. Subsequently, an epinephrine infusion, titrated up to 0.07 mcg/kg/min, was started. The patient's hemodynamics did not improve and deterioration of liver function was noted. Cholangitis from stricture formation was suspected and the patient underwent a bedside endoscopic retrograde cholangiopancreatography (ERCP) with stent placement in the common bile duct.

After ERCP, the patient's condition continued to deteriorate. He remained febrile and hemodynamically unstable, with a heart rate of 137 bpm and BP of 87/55 mmHg despite support of multiple pressors and inotropes. Urine output decreased and serum creatinine rose to over 3 mg/dL. Acute kidney failure developed requiring continuous renal replacement therapy (CRRT). In order to counteract a presumed suppression of the adrenal hypothalamic axis, intermittent boluses of hydrocortisone and a levothyroxine infusion (30 mcg/h) were started.

**Figure 1:**
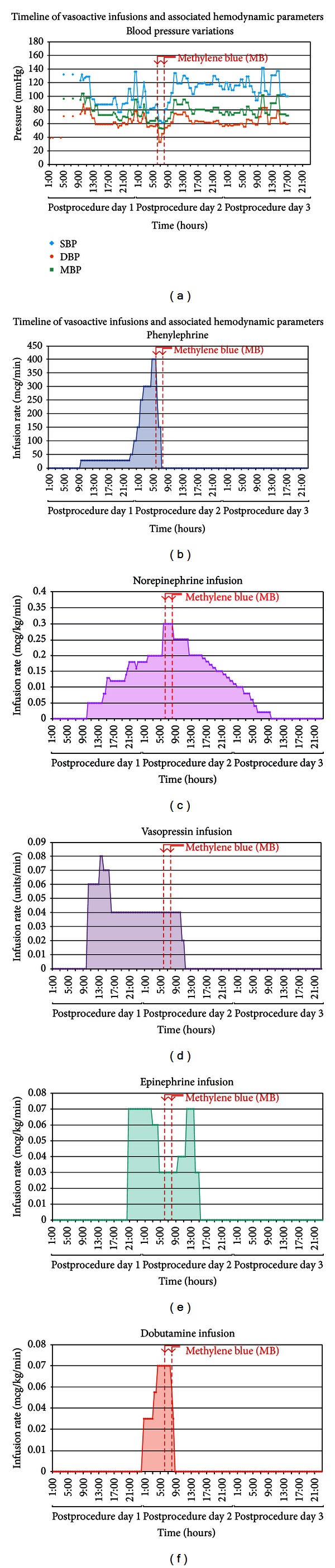
(a) Graph showing blood pressure variations across time period; blood pressure (Blue—SBP; brown—DBP; green—MAP) in *Y* axis and time elapsed in hours from postprocedure day 1 to post procedure 3 in *X* axis. (b) Graph showing phenylephrine infusion with infusion rate (mcg/min) in *Y* axis and time elapsed in hours from postprocedure day 1 to postprocedure 3 in *X* axis. (c) Graph showing norepinephrine infusion with infusion rate (mcg/kg/min) in *Y* axis and time elapsed in hours from postprocedure day 1 to postprocedure 3 in *X* axis. (d) Graph showing vasopressin infusion with infusion rate (Units/min) in *Y* axis and time elapsed in hours from postprocedure day 1 to postprocedure 3 in *X* axis. (e) Graph showing epinephrine infusion with infusion rate (mcg/kg/min) in *Y* axis and time elapsed in hours from postprocedure day 1 to postprocedure 3 in *X* axis. (f) Graph showing dobutamine infusion with infusion rate (mcg/kg/min) in *Y* axis and time elapsed in hours from postprocedure day 1 to postprocedure 3 in *X* axis. Double dash vertical lines across graphs (a, b, c, d, e and f) depict the period of methylene blue (MB) infusion.

On PPD 2, after exhausting all therapeutic measures and with further deterioration of the patient's condition, MB therapy was initiated with two 100 mg doses (2 mg/kg) infused over a period of 2 hours. Three hours after administration of the first dose of MB, the patient's hemodynamic parameters markedly improved (BP of 117/62, HR of 117, SVR increased to 536 dyne·s^−1^·cm^−5^, and mixed venous saturation decreased to 60%). These changes reflect recovery of systemic microperfusion with subsequent increase in oxygen consumption and a reduction of arterio-venous shunting. The patient was subsequently weaned from vasoactive support (Figure 1) and mechanical ventilation was discontinued 3 days later. The patient's urine output markedly improved, CRRT was stopped, and he was transferred to the ward. The patient was discharged home on PPD 12.

## 3. Discussion 

There are a number of publications demonstrating effectiveness of MB for treatment of sepsis-associated vasoplegia. This paper is the first known documentation of the therapeutic effects of MB for treatment of severe sepsis in an *immunocompromised *patient. The treatment of sepsis in immunosuppressed patients is one of most difficult challenges in modern critical care. The unbalanced inflammatory reaction, frequently seen in septic immunosuppressed patients, produces massive vasodilatation with subsequent severe injury to organ microperfusion.

Sepsis itself is associated with significant immune dysregulation. Defects in the function of leukocytes, alterations in antigen-presenting ability, and accelerated apoptosis contribute to the systemic inflammatory response which results in the release of cytokines and endotoxins [5, 6]. Endotoxins directly initiate production of inducible NO synthase (iNOS) [7–9]. In addition, they activate release of interleukin 1-b, tumor necrosis factor-*α*, and interferon-*γ*. These cytokines are responsible for increasing iNOS release in target cells, which leads to an elevation in NO production from L-arginine in vascular endothelium [10]. NO, in turn, activates guanyl-cyclase (GC) with subsequent increasing concentrations of cGMP in vascular smooth muscle. This results in systemic vasodilation. 

There is a significant difference in the immune response to sepsis between the immunocompetent and immunocompromised patient. Immunocompetent patients usually demonstrate a specific *anti-inflammatory* reaction as an immediate response to sepsis in order to localize the inflammatory process and prevent diffuse vascular damage [11]. This reaction includes release of various cytokines including interleukin-1 and interleukin-10 receptor antagonists as well as soluble tumor necrosis factor receptor. These substances inhibit the T-cell network with subsequent downregulation of monocyte activation. In addition, the development of tolerance to lipopolysaccharide (a major constituent of gram negative bacterial cell walls) as well as cellular deactivation of macrophages and monocytes is part of this protective process [12].

This anti-inflammatory mechanism is severely depressed in immunocompromised patients which results in an uncontrolled inflammatory response with subsequent hyper-resistant hemodynamic instability [13]. It has been demonstrated that immunosuppressed septic patients are prone to develop septic shock and demonstrate an even more refractory hypotension in comparison to patients without immunosuppression [14]. 

The main cause of this vasoplegia is the overpowering activation of the NO pathway due to an unbalanced systemic inflammatory reaction. Administration of MB has been demonstrated to reverse the course of hemodynamic deterioration and prevent development of severe septic shock.

MB acts on multiple sites in the cGMP pathway. Specifically, MB inhibits iNOS in vascular endothelium which effectively reduces NO production [15]. MB also binds soluble GC which results in a reduction in cGMP levels [16, 17]. Additionally, it has been demonstrated that MB contributes to increasing cardiac contractility depressed by cGMP [18].

Severe sepsis is associated with a mortality rate over 40% [18]. Sepsis-associated mortality in immunosuppressed patients is even higher. Poutsiaka et al. [14] reviewed the outcome of immunosuppressed patients with severe sepsis. They demonstrated significantly higher 28-day mortality in patients with preexisting immunosuppression in comparison with immunocompetent septic patients. 

The main cause of sepsis associated mortality is multiorgan failure related to breakdown of tissue microcirculation. Considering that organ perfusion in the immunosuppressed septic patient is more severely affected than in the immunocompetent septic patient due to the uncontained inflammatory response, early administration of MB can be beneficial.
